# Mapping the future of early breast cancer diagnosis: a bibliometric analysis of AI innovations

**DOI:** 10.1007/s12672-025-03495-y

**Published:** 2025-10-14

**Authors:** Şevki Pedük

**Affiliations:** Surgical Oncology, Balıkesir Atatürk City Hospital, No: 26, 209 Street, Gaziosmanpasa Neighborhood Altieylul District, Balıkesir, 10100 Turkey

**Keywords:** Artificial intelligence, Bibliometric analysis, Breast cancer, Clinical collaboration, Deep learning

## Abstract

Breast cancer (BC) remains one of the most prevalent and challenging malignancies worldwide, affecting millions of women and shaping healthcare priorities across continents. Advances in early detection have significantly improved survival rates. In recent years, artificial intelligence (AI) has emerged as a powerful tool in this domain, transforming traditional diagnostic methods. Initially based on simple rule-based systems, AI has evolved into sophisticated deep learning models capable of analyzing complex medical data with remarkable accuracy. This bibliometric analysis examines the application of AI in the early diagnosis of breast cancer, aiming to understand not only the current state of the field but also its growth over the past decade. Publications indexed in Web of Science and Scopus from 2012 to March 2025 were systematically reviewed, while earlier literature (1994–2012) provided historical context. Tools such as Biblioshiny and VOSviewer were used to map research trends, collaboration patterns, and thematic evolution. Out of 1,436 initial documents, 1,293 high-quality studies were included. The results show a clear acceleration in AI-focused research after 2020, with increased global collaboration and a notable shift toward open-access publication. Recurring themes such as “machine learning,” “diagnostic imaging,” and “clinical decision support” highlight the field’s direction. As AI becomes more integrated into clinical workflows, its potential to enhance diagnostic speed, consistency, and personalization is undeniable. However, key ethical issues such as bias, transparency, and patient data protection remain central to responsible implementation.

## Introduction

Breast cancer (BC) is a manageable disease influenced by genetic, hormonal, environmental, nutritional, age-related, and lifestyle factors. Protective elements such as multiparity, breastfeeding, and regular physical activity have been well documented [1]. In the United States, one in eight women is expected to develop breast cancer during their lifetime. Although this rate varies across regions, it highlights BC as a major global health issue [2]. According to epidemiological data, the age-adjusted incidence in women is nearly 130 per 100,000, compared to only 1–2 cases per 100,000 in men [3]. Broader access to imaging has contributed to higher detection rates, while mortality has steadily declined and is currently around 20 per 100,000 [4].

In developed countries, structured screening programs have improved survival and reduced healthcare burden. At the same time, artificial intelligence (AI) is increasingly being applied to healthcare, especially in early detection, where it shows strong potential [5].

AI in medicine has advanced in several phases. In the 1970s, early expert systems such as MYCIN offered rule-based clinical decision support, but their rigid design limited use in practice [6]. The 1980s brought the backpropagation algorithm, enabling multilayer neural networks to learn from data [7]. In the 1990s, support vector machines (SVMs) and Bayesian models advanced pattern recognition and supported the first computer-aided diagnostic (CAD) systems. In breast cancer, researchers like Giger and Karssemeijer developed algorithms to detect microcalcification clusters in mammograms, among the earliest AI applications for early diagnosis [8].

The past decade has been defined by the growth of deep learning (DL), fueled by big data and stronger computational resources. Convolutional neural networks (CNNs) now underpin much of medical image analysis, improving diagnostic accuracy and speeding up clinical adoption. This historical path—from expert systems to deep learning—sets the framework for the present study, which uses bibliometric methods to map how AI in early breast cancer diagnosis has evolved and where it is heading.

AI is now widely applied across industries, including healthcare, where it plays a central role in analyzing medical images. The use of AI in early BC detection has become a growing focus of research. Bibliometric studies provide a systematic way to track how this field has developed, highlighting trends, collaborations, and emerging themes [9]. Yet, important gaps remain in the literature.

Although many studies have explored AI in breast cancer research, few have focused specifically on early diagnosis. Earlier bibliometric analyses have often relied on a single database, covered shorter time periods, or lacked structural mapping techniques. In contrast, this study integrates data from both Web of Science and Scopus, examines the period from 2012 to the first quarter of 2025—while incorporating earlier literature (1994–2011) for context—and focuses uniquely on early-stage diagnosis. By applying descriptive, conceptual, and relational bibliometric approaches, the analysis offers a comprehensive and clinically relevant view of how the field has progressed, as presented in the following sections.

## Methods

In this study, the evolution of the field was examined along with current trends by reviewing academic publications on AI applications in the early diagnosis of BC through a bibliometric lens. The literature review was based on the Web of Science (WOS) and Scopus databases. Data from both databases were exported in appropriate formats and merged using common fields through the Biblioshiny software, an open-source tool developed for bibliometric analysis in R Studio [10]. The year 2012 was selected as the starting point because the number of articles had doubled compared to the previous year. However, 45 earlier publications, dating from 1994 (when the first relevant article appeared) to 2012, were also considered to provide historical context and track the evolution of the field.

### Data source

WOS and Scopus databases were used as data sources. These databases were selected due to their comprehensive interdisciplinary coverage and high indexing standards, making them the most widely used sources for bibliometric research. Additionally, they provide well-structured metadata and standardized export formats, ensuring full compatibility with bibliometric analysis tools such as Biblioshiny and VOSviewer. Although other databases like PubMed, IEEE Xplore, ScienceDirect, DOAJ, and JSTOR contain relevant literature, focusing primarily on WOS and Scopus allowed us to maintain consistency and comparability across the dataset. Only articles, proceedings papers, review articles, and early access publications were included. The quality of the research data was ensured by applying strict inclusion criteria.

### Data extraction & data collection

The search strategy employed keywords related to BC (“breast cancer,” “mammary carcinoma,”) and AI (“artificial intelligence,” “machine learning,” “deep learning,” “computational intelligence,” “neural networks”). To narrow the focus to early diagnosis, the terms “early diagnosis,” “early detection,” and “early screening” were also included in the query. The “Full Record and Cited References” export option was used to ensure that metadata such as authorship, publication year, journal title, citation count, and institutional information were fully captured.

The full Boolean search string used was: (“breast cancer” OR “mammary carcinoma”) AND (“artificial intelligence” OR “machine learning” OR “deep learning” OR “computational intelligence” OR “neural networks”) AND (“early diagnosis” OR “early detection” OR “early screening”).

### Exclusion criteria

In addition to removing duplicate records, publications classified as editorials, letters, comments, meeting abstracts, corrections, errata, retractions, book chapters, and books were excluded from the analysis.

### Analysis and mapping

For data analysis, plain text files were processed using Biblioshiny (ver. 2024.12.1–563). This allowed identification of major trends in publication output and patterns of collaboration between authors and countries. Co-authorship and co-word analyses were performed to explore these relationships in more detail.

Additionally, VOSviewer (ver. 1.6.20; Center for Science and Technology Studies, Leiden University) was used with tab-delimited files to map collaboration networks and conceptual structures [11].

This approach enables a comprehensive evaluation of current practices by systematically revealing the role of AI applications in the literature, their development over time, and collaborative dynamics among researchers in the early diagnosis of breast cancer.

## Results

### Descriptive analysis

#### Annual distribution of publications

**Fig. 1:**
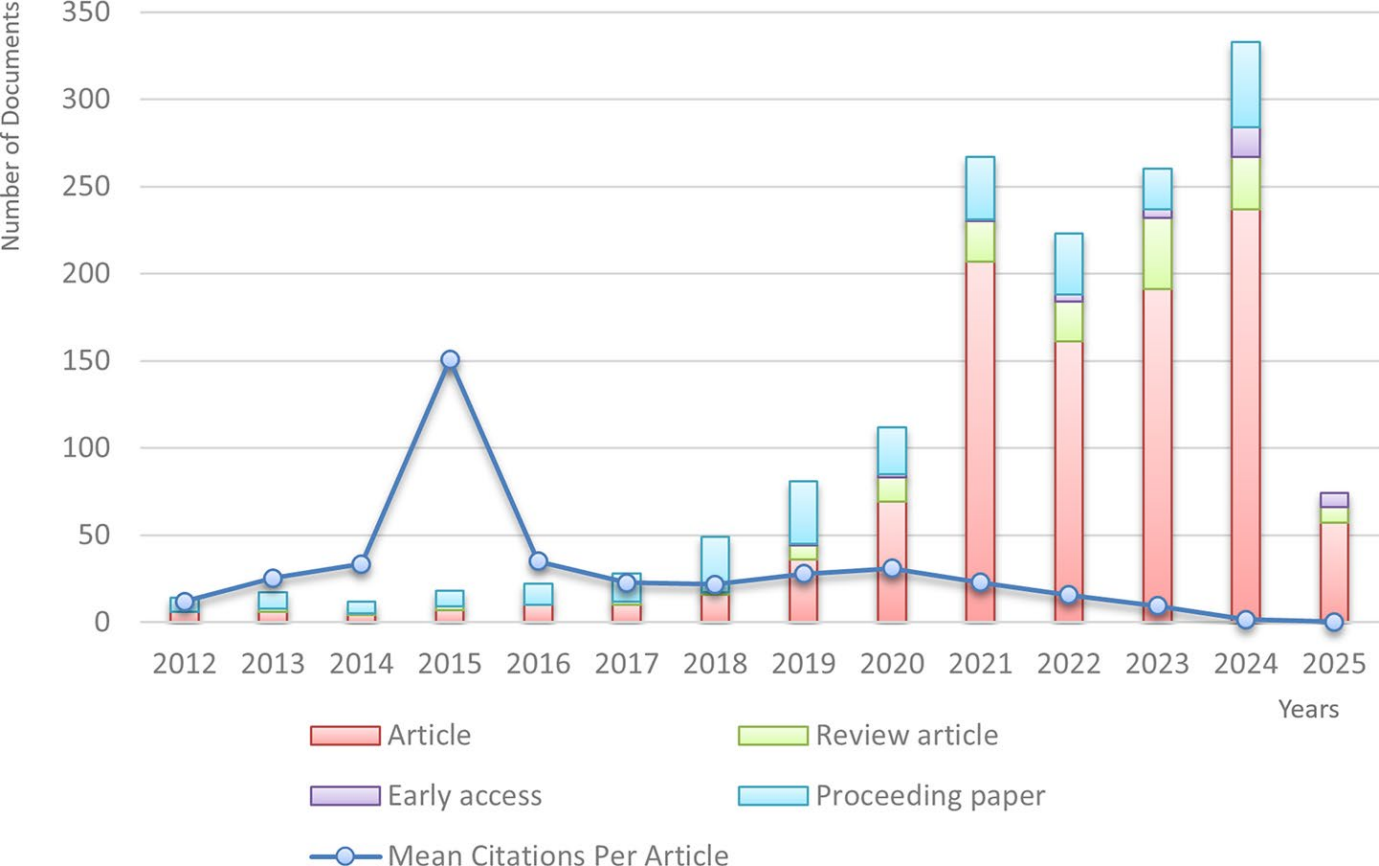
Distribution of publications and citations

A total of 1,436 documents were retrieved from the WOS and Scopus databases. After excluding those that did not meet the quality criteria, 1,408 articles remained. As the annual number of publications stayed below ten until 2012, our analysis focused on the 1,363 articles published between 2012 and 2025. After removing duplicates, the remaining 1,293 articles from 667 journals constituted the core dataset for analysis. Of these, 643 articles (approximately 50%) were open access (OA), indicating that nearly half of the literature is freely accessible. Among these, the majority were Gold OA (469), followed by Green Published (291), Gold Hybrid (84), Free-to-Read (36), Green Accepted (22), and Green Submitted (58). From 2012—when 11 articles were published—until 2017, publication output remained low and relatively stable. A notable increase occurred in 2018–2020, with the number of publications surpassing the 250-mark in 2021. The field peaked in 2024 with 320 publications. As of the first quarter of 2025, 77 articles have already been published, suggesting continued growth. The overall average annual growth rate is 17.95%. Original research articles began to dominate in 2020. The dataset received a total of 20,518 citations (18,452 excluding self-citations), indicating growing scholarly interest in AI applications for early BC diagnosis and the presence of a robust reference base. The distribution of article types and citations by year is shown in Fig. 1.

#### Scientific production of countries

**Fig. 2:**
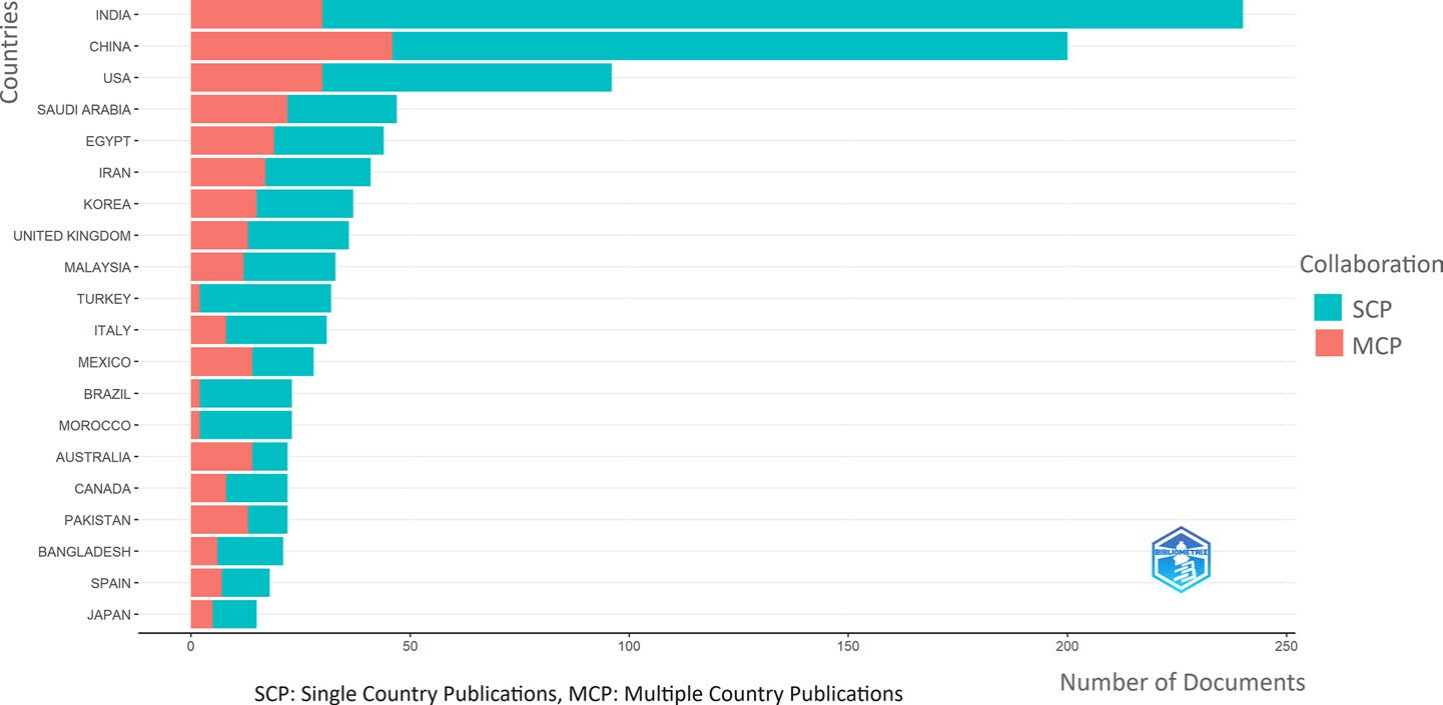
Most relevant countries

When only corresponding authors are considered for attributing a publication to a single country, India ranks first with 240 articles, followed by China (205) and the United States (97). While single-country publications (SCPs) dominate in all countries, the rate of multi-country collaboration (MCP) is notably higher in China than in other leading nations (Fig. 2). The overall international collaboration rate is 29.11%, underscoring the interdisciplinary and cross-border importance of this research field.

Country-level collaboration patterns reveal that research activities are particularly concentrated in Asia, North America, and Western Europe, reflecting the global relevance of AI applications in breast cancer diagnosis.

According to the time-series analysis, a sharp increase in global article production has been observed since 2020. By 2024, the combined output of India and China exceeded 1,200 publications. In terms of citation performance, China (3,494) and India (2,364) lead in total citations, while Greece (1,772) stands out for its average citations per publication. The United States maintains a prominent position with both high productivity (1,717 total citations) and a strong average citation impact (17.7) (Table 1).

**Table 1:** Production and citation volumes of countries

Country	G. Cit.	Av. Doc. Cit.	Cor. Au. Cou.	Cou. Sci. Pro.
CHINA	3,494	17.00	205	662
INDIA	2,364	9.80	240	618
GREECE	1,772	221.50	8	28
USA	1,717	17.70	97	345
EGYPT	1,171	27.20	43	129
IRAN	1,027	25.00	41	137
MALAYSIA	898	26.40	34	95
TURKEY	615	19.20	32	69
KOREA	583	16.20	36	115
PAKISTAN	526	23.90	22	112

*G. Cit.* Global Citations, *Av. Doc. Cit.* Average Document Citations, *Cor. Au. Cou.* Corresponding Author Country, *Cou. Sci. Pro.* Country Scientific Production.

#### Funding organizations and countries

About 55% of the studies did not report any funding source. Among those that did, China accounted for the highest number of funded publications (120, 8.78%), followed by the United States (68, 4.97%) and South Korea (39, 2.85%). The most frequently mentioned funding agency was the National Natural Science Foundation of China (NSFC), which supported 101 publications. Both the National Institutes of Health (NIH) and the U.S. Department of Health and Human Services were listed as funders in 27 studies each.

#### Institutional production

Based on data retrieved through the Web of Science interface, the most frequently listed institutions were the Egyptian Knowledge Bank (EKB) with 64 publications, the National Institute of Technology with 23, and the Vellore Institute of Technology with 19. However, since organizations like the EKB function as national publication platforms rather than traditional research institutions, a more detailed analysis was carried out using departmental-level affiliations. This closer look revealed a different ranking: Princess Nora Bint Abdul Rahman University’s College of Computer and Information Sciences led with 13 publications, followed by the Faculty of Computing and Information Technology at King Abdulaziz University (8), and the College of Computer and Information Sciences at King Saud University (7).

**Fig. 3:**
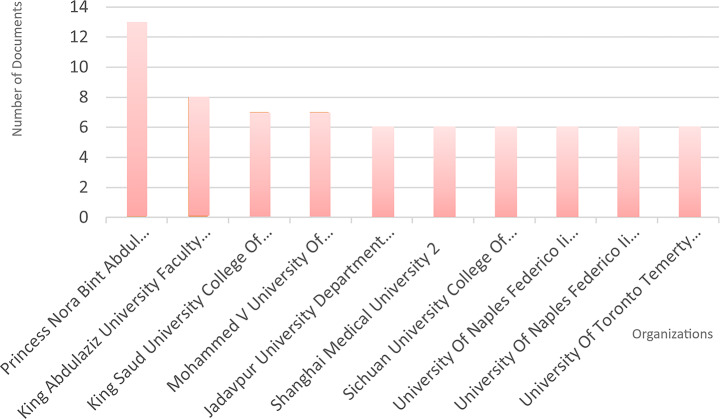
Most productive organizations

While the Chinese Academy of Sciences received the highest number of citations (624), Princess Nora Bint Abdul Rahman University had the strongest total link strength (17). Figure 3 illustrates the most productive institutions. Co-authorship patterns suggest that collaboration between institutions remains relatively limited, with only a few closely connected groups showing strong internal ties.

#### Distribution of journals

**Fig. 4:**
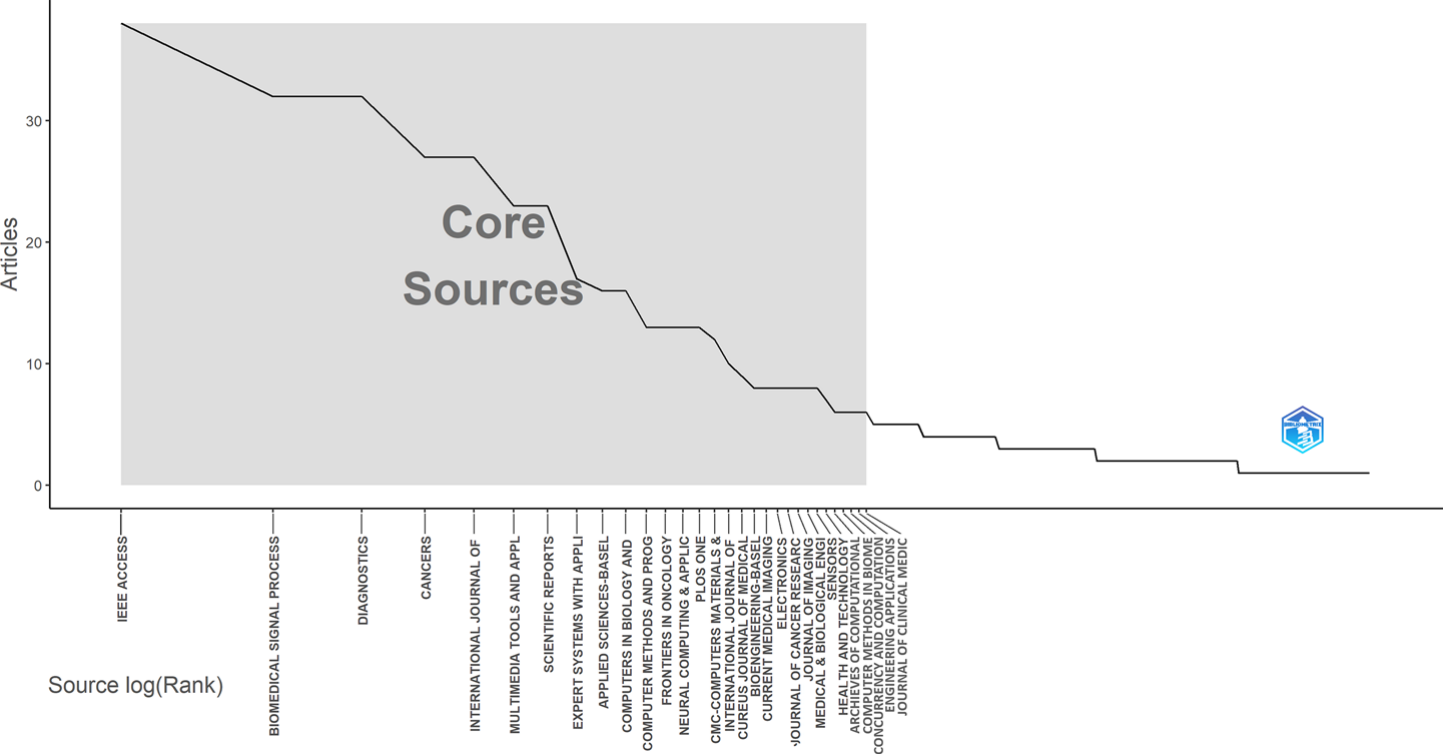
Bradford’s law

As the overall number of publications in the field has grown, journal outputs have also increased—particularly in IEEE Access, which focuses on information technology, and Cancers, a journal known for its emphasis on clinical applications. Since 2020, both have shown a noticeable rise in publication volume. Among the 667 journals identified, IEEE Access leads with 38 articles, followed by Biomedical Signal Processing and Control (33) and Diagnostics (32). Other notable journals include Cancers (28), International Journal of Advanced Computer Science and Applications (27), and Multimedia Tools and Applications (24). These journals also fall within the “core sources” defined by Bradford’s Law, which categorizes journals into core and peripheral zones based on their productivity in a given research area [12]. Figure 4 presents the distribution of these key journals and their roles within the field.

In terms of local citations, IEEE Access ranks first with 925 citations, followed by Lecture Notes in Computer Science (888) and Expert Systems with Applications (880). Diagnostics stands out with its high average citation performance (h-index 15) and rapidly growing publication trend (Table 2). Co-citation patterns show thematic clustering among a small number of journals, indicating that research in this area is shaped by a few key publication sources.

**Table 2:** Publication volumes, citation performance, and journal impact

Source	h-index	g-index	m-index	G. Cit.	Doc.	Cit. Per Doc.	F. Pub.Y.	İ. F.
Diagnostics	15	25	1.667	672	32	21.00	2017	3
Computers in biology and medicine	14	16	1.000	703	16	43.94	2012	7.7
IEEE access	13	29	1.857	882	38	23.21	2019	3.4
Expert systems with applications	12	17	0.857	1,286	17	75.65	2012	7.5
Biomedical signal processing and control	9	18	1.800	358	32	11.19	2021	4.1
Cancers	9	14	1.800	233	27	8.63	2021	5.2
Computer methods and programs in biomedicine	7	13	1.167	243	13	18.69	2020	4.9
International journal of advanced computer science and applications	7	13	0.700	184	27	6.81	2016	0.7
Multimedia tools and applications	7	14	1.167	215	23	9.35	2020	3
Neural computing & applications	7	13	1.400	228	13	17.54	2021	4.5

*G. Cit.* Global Citations, *Doc.* Document, *Cit. Per Doc.* Citations Per Document, *F. Pub. Y.* First Publication Year, *I. F.* Impact Factor.

#### Distribution of authors

**Fig. 5:**
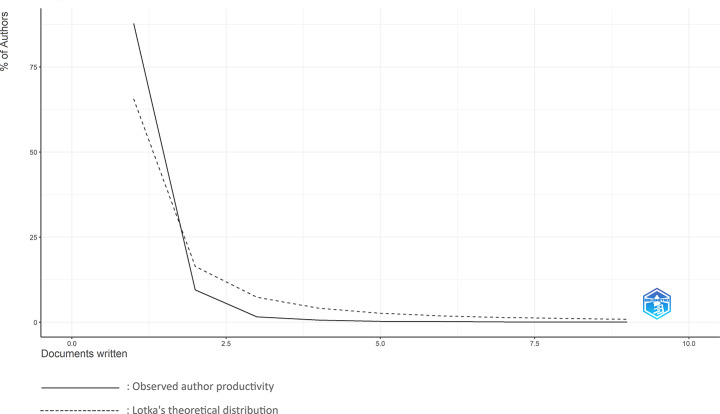
Author productivity distribution based on Lotka’s Law The solid line represents the observed number of authors by publication count, while the dashed line indicates the expected distribution according to Lotka’s theoretical model

Among the 1,293 articles analyzed, 43 (3.3%) were written by a single author. A total of 5,351 researchers contributed to the field, with an average of 4.84 authors per article. Only 29 authors (0.54%) published five or more papers, while 87.8% contributed just once. This distribution reflects the early development of the field and aligns with Lotka’s Law, which states that the number of authors publishing *n* papers is roughly 1/*n*² of those publishing one [13]. Figure 5 illustrates this trend.

Table 3 provides key data on author productivity and impact. Although Khan M.A. was the most productive author with nine articles, only three had strong connections in the co-author network, suggesting that productivity alone may not lead to greater influence in the field.

The 2015 review article titled “Machine Learning Applications in Cancer Prognosis and Prediction” received 1,726 citations, making it the most influential study to date. As a result, Exarchos T.P. (2 articles) and four co-authors (1 article each) rank among the top five most cited authors (Table 3). This highlights how a single impactful publication can shape the field.

Based on annual publication and citation data, Sarkar R., Yang J., and Li J. stand out with high h-index values and strong citation rates per article. Although most of Luca Nicosia’s work focuses on radiology and oncology, his high m-index (1.667) reflects recent concentrated research activity. While only part of his work directly addresses AI in early breast cancer diagnosis, his contributions to breast imaging and his role in a closely connected author network indicate growing influence in this interdisciplinary area.

**Fig. 6:**
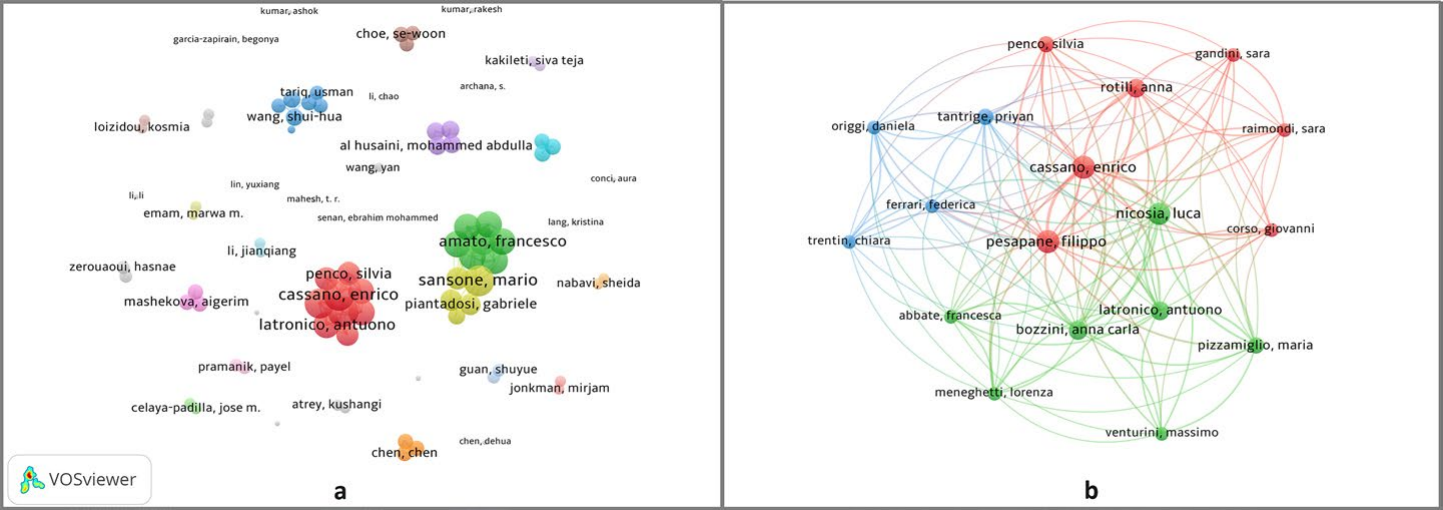
Co-authorship analysis (**a**) Global co-authorship network of 469 authors with ≥ 2 co-authored documents (**b**) Zoomed-in view of the most cohesive cluster of 18 authors

Figure 6a shows the global co-authorship network of 469 authors with at least two joint publications. Node size and proximity reflect collaboration strength. Figure 6b highlights the most cohesive cluster of 18 authors, with Pesapane F. (87), Cassano E. (87), and Nicosia L. (78) having the highest total link strengths. Their strong internal ties suggest a central role in collaboration and knowledge sharing.

**Table 3:** Evaluating author productivity and scholarly impact

Author	Document	G. Cit.	Cit. / Doc.	h-index	m-index	F. Pub. Y.	T. Link Str.
A Authors with the highest h-index							
SARKAR R	7	186	26.57	6	1.200	2021	40
CASSANO E	7	106	15.14	5	0.833	2020	87
DAS A	5	101	20.2	5	0.833	2020	2
KHAN MA	9	168	18.66	5	1.000	2021	24
LI J	6	278	46.33	5	0.625	2018	8
NICOSIA L	6	69	11.5	5	1.667	2023	78
PESAPANE F	7	106	15.14	5	0.833	2020	87
SANSONE M	7	148	21.14	5	0.556	2017	40
WANG SH	6	93	15.50	5	1.000	2021	18
YANG J	5	357	71.40	5	0.625	2018	8
B Authors with the highest citation							
EXARCHOS TP	2	1,727	863.50	1	0.091	2015	6
EXARCHOS KP	1	1,726	1,726	1	0.091	2015	4
FOTIADIS DI	1	1,726	1,726	1	0.091	2015	4
KARAMOUZIS MV	1	1,726	1,726	1	0.091	2015	4
KOUROU K	1	1,726	1,726	1	0.091	2015	4

*G. Cit*. Global Citations, *Cit./Doc.* Citations Per Document, *F. Pub. Y.* First Publication Year, *T. Link Str.* Total Link Strength.

#### Document impact analysis

Table 4 lists the 20 most cited studies on AI applications in early breast cancer diagnosis. At the top is the 2015 article by Kourou K., with 1,726 global citations, 40 local citations, 156.9 citations per year, and a normalized score of 11.4. Its high citation count may be related to its format as a review article, open access availability, and the timing of its publication—when machine learning was just gaining momentum—along with a strong methodological approach.

The second most cited paper is by Huang S.G. (2020), published in *Cancer Letters*, with 313 global citations. This supports the idea that review articles continue to play a key role in summarizing new developments and helping disseminate them quickly across the scientific community.

Meanwhile, original research articles like the one by Abdelzaher A.M. (2016), published in *Expert Systems with Applications*, have also made an impact. With 283 global citations, 29 local citations, 28.3 citations per year, and a normalized score of 8.06, this study shows that high-quality empirical research also contributes meaningfully to the field. Similarly, studies by Rouhi R. (2015) and Jalalian A. (2013) demonstrate how both original and review articles shape the evolving research landscape.

Recent publications are also making a mark. For instance, Houssein E.H. (2021) has 198 global citations and an average of 39.6 citations per year. Even more striking is the 2023 article by Lång K., which, despite having 173 global citations, has a higher annual rate (57.67) and a normalized impact of 17.73. These figures highlight how newer research, supported by advanced techniques and growing interest, can achieve rapid recognition.

Articles by Saber A., Ahsan M.M., Dembrower K., and Dlamini Z. have also gained considerable attention, likely due to being open access. Overall, the combination of global and local citations, annual averages, and normalized impact in Table 4 offers a broad view of how publication type, accessibility, and timing influence scholarly impact in this fast-moving field.

**Table 4:** Document impact analysis the top 20 most cited documents globally

Document	DOI	G.Cit.	L. _Cit_.	Cit. / _Year_	Nor. Cit.	Type	O. A.
KOUROU K, 2015, COMPUT STRUCT BIOTEC	10.1016/j.csbj.2014.11.005	1726	*40	156.91	11.43	Review	+
HUANG SG, 2020, CANCER LETT	10.1016/j.canlet.2019.12.007	313	10	52.17	10.07	Review	
ABDEL−ZAHER AM, 2016, EXPERT SYST APPL	10.1016/j.eswa.2015.10.015	283	**29**	28.30	8.06	Article	
ROUHI R, 2015, EXPERT SYST APPL	10.1016/j.eswa.2014.09.020	272	**18**	24.73	1.80	Article	
JALALIAN A, 2013, CLIN IMAG	10.1016/j.clinimag.2012.09.024	237	13	18.23	9.33	Review	
LIU R, 2014, MED RES REV	10.1002/med.21293	220	0	18.33	6.59	Review	
HOUSSEIN EH, 2021, EXPERT SYST APPL	10.1016/j.eswa.2020.114161	198	*32	39.60	8.62	Review	
LÅNG K, 2023, LANCET ONCOL	https://doi.org/10.1016/S1470−2045(23)00298−X	173	8	57.67	17.73	Article	
SABER A, 2021, IEEE ACCESS	10.1109/ACCESS.2021.3079204	168	*31	33.60	7.31	Article	+
AHSAN MM, 2022, HEALTHCARE−BASEL	10.3390/healthcare10030541	167	1	41.75	10.56	Article	+
DEMBROWER K, 2020, LANCET DIGIT HEALTH	NA	151	0	25.17	4.86	Article	+
DLAMINI Z, 2020, COMPUT STRUCT BIOTEC	10.1016/j.csbj.2020.08.019	150	3	25.00	4.83	Review	+
ABDELHAFIZ D, 2019, BMC BIOINFORMATICS	https://doi.org/10.1186/s12859−019−2823−4	147	**19**	21.00	5.34	Proceeding	+
KEMP JA, 2021, NANO CONVERG	https://doi.org/10.1186/s40580−021−00282−7	145	0	29.00	6.21	Review	+
XIE JY, 2019, FRONT GENET	10.3389/fgene.2019.00080	144	**20**	20.57	5.23	Article	+
MONTAZERI M, 2016, TECHNOL HEALTH CARE	https://doi.org/10.3233/THC−151071	142	7	14.20	4.05	Article	+
BHARDWAJ A, 2015, EXPERT SYST APPL	10.1016/j.eswa.2015.01.065	140	**22**	12.73	0.93	Article	
GU DX, 2017, INT J MED INFORM	10.1016/j.ijmedinf.2016.11.006	130	1	14.44	5.70	Review	
WANG JH, 2016, SCI REP−UK	10.1038/srep27327	128	16	12.80	3.65	Article	+
THAKUR V, 2019, J EXP CLIN CANC RES	https://doi.org/10.1186/s13046−019−1443−1	128	9	18.29	4.65	Review	+

*G. Cit.* Global citations, *L. Cit.* Local citations, *Cit. / Year* Citations Per Year, *Nor. Cit.* Normalized Citations, *O. A.* Open Access.

Bold: Local Citations top 20.

*Local Citations top 3.

### Conceptual structure analysis

#### Tree field plot

**Fig. 7:**
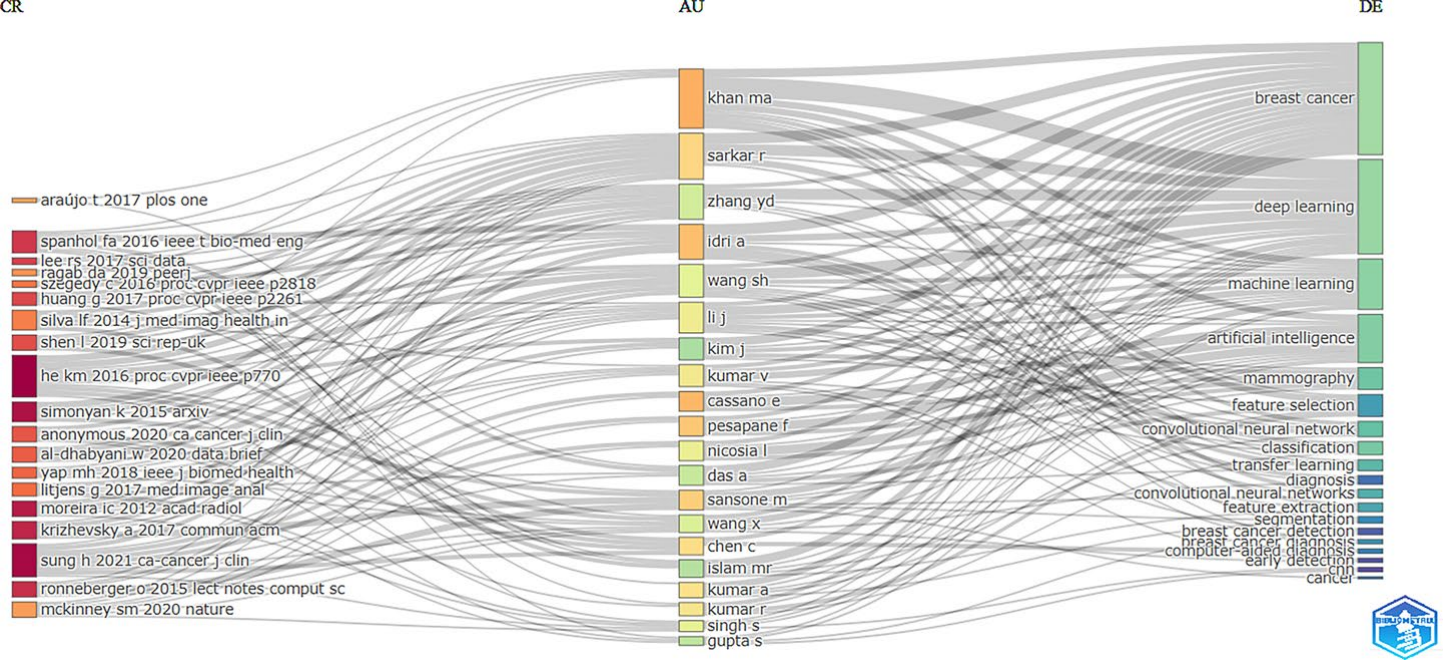
Tree-field plot

Figure 7 presents a tree-field plot that visually connects cited references (CR), authors (AU), and keywords (DE) in the research field. Prominent authors such as Khan M.A., Idri A., Cassano E., Pesapane F., and Sansone M. tend to focus on keywords like “breast cancer,” “deep learning,” “artificial intelligence,” “machine learning,” and “classification.”

The most frequently cited works include He K.M.’s paper on the ResNet architecture [14], Sung HY.’s study on optimizing deep learning methods [15], and Simonyan K.’s work on image classification [16].

#### Co-occurrence network & thematic map

**Fig. 8:**
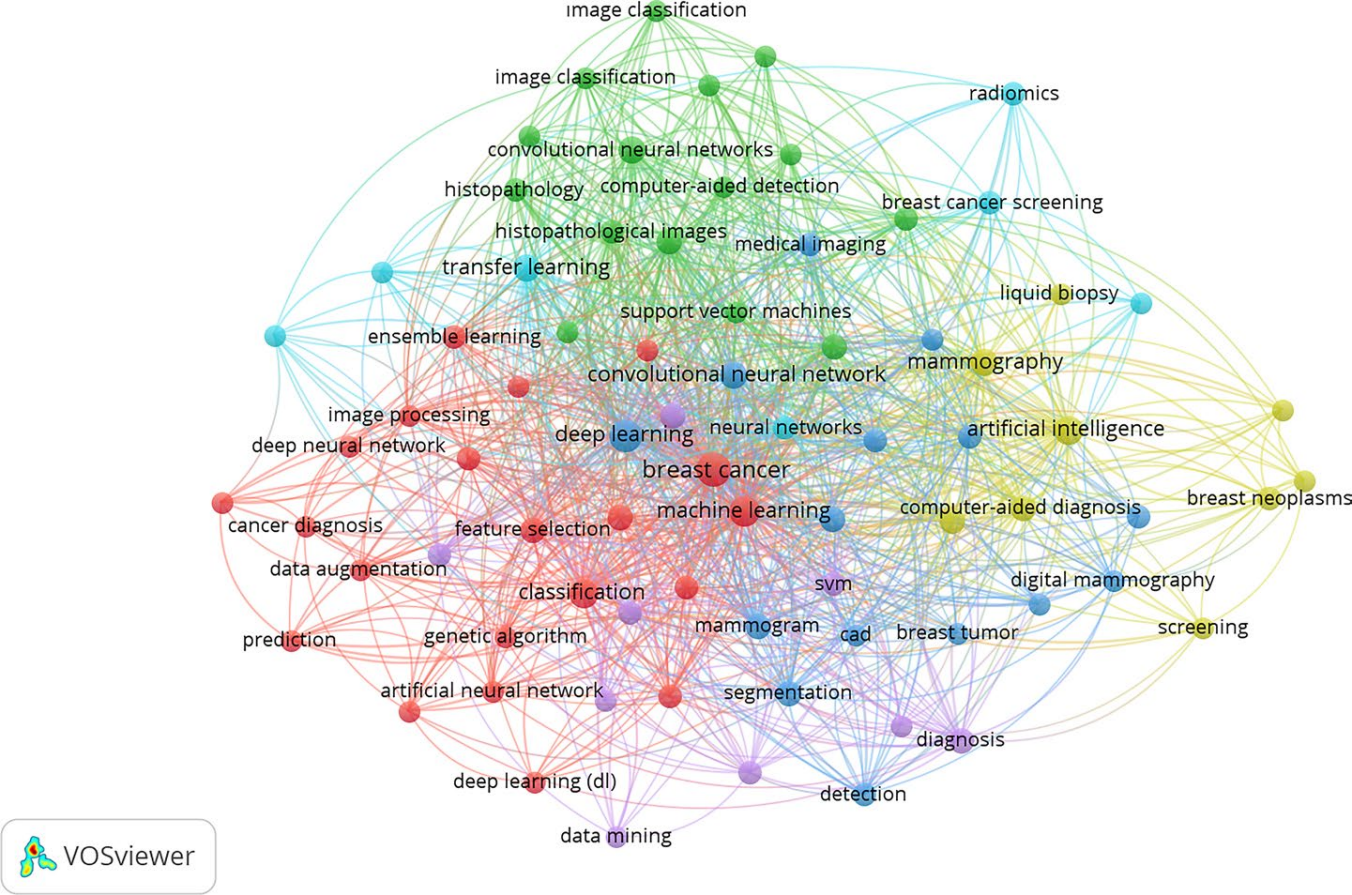
Co-occurrence keyword analysis network

The co-occurrence keyword analysis generated a clear visual map using a minimum threshold of 10 keyword appearances (Fig. 8). At the center of the network is “breast cancer,” closely linked with key methodological terms such as “machine learning,” “deep learning,” and “convolutional neural network.” These, in turn, connect to concepts like “classification,” “support vector machines,” “computer-aided diagnosis,” and “mammogram,” forming the conceptual core of the field. Emerging topics such as “radiomics,” “magnetic resonance imaging,” and “liquid biopsy” also appear but are located at the periphery, reflecting their growing yet still secondary role. Although not shown here, overlay visualizations indicate that these terms have become more prominent in recent years.

**Fig. 9:**
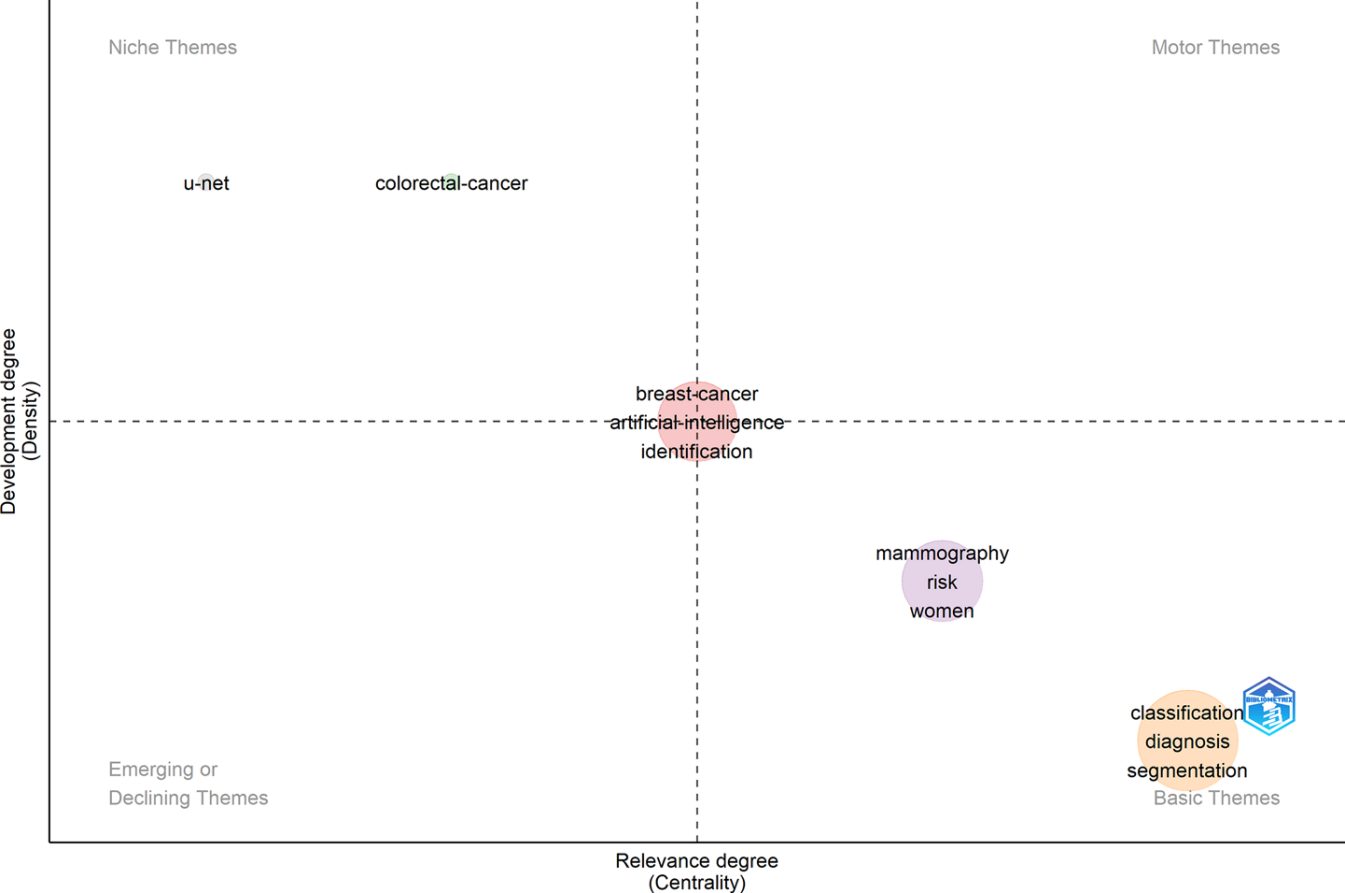
Thematic map

In support of this analysis, the thematic map (Fig. 9) organizes keyword clusters along two dimensions: centrality (their importance within the broader research field) and density (their internal development). As illustrated, “breast cancer,” “artificial intelligence,” and “diagnostics” appear near the center, representing well-developed and influential themes. In contrast, terms like “classification,” “diagnosis,” and “segmentation” are positioned in the lower-right quadrant, suggesting basic but widely used concepts. Meanwhile, themes such as “colorectal cancer” are found in the upper-left quadrant, representing well-developed and specialized research areas. In contrast, “U-Net,” which is a specific deep learning model rather than a thematic research area, is categorized as a methodological niche. This structure offers a clear overview of both foundational topics and emerging directions in the literature.

#### Thematic evolution

**Fig. 10:**
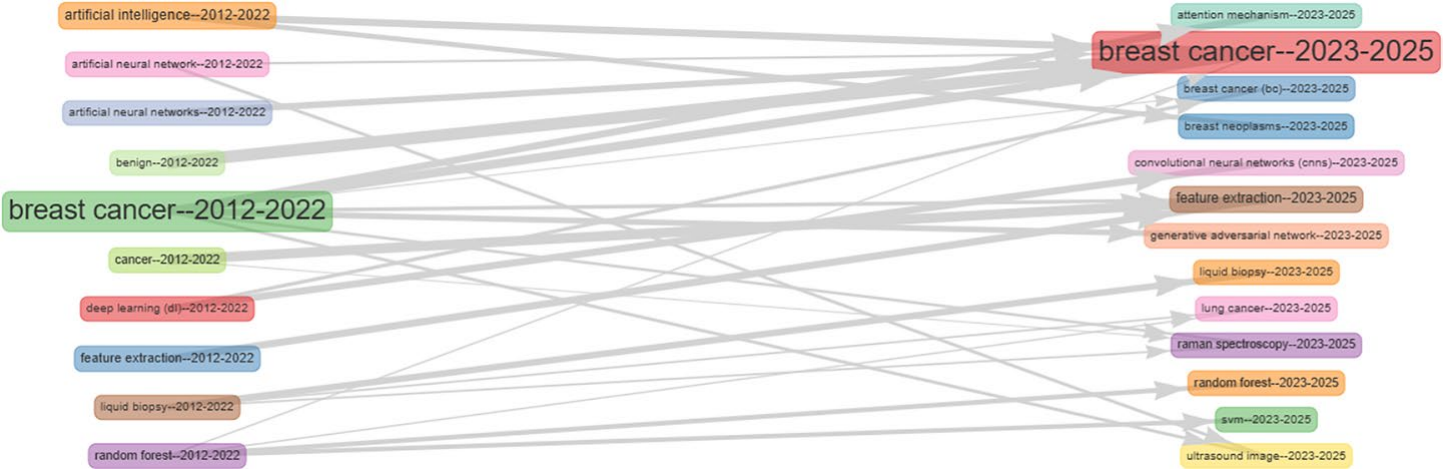
Thematic evolution

Figure 10 illustrates the thematic evolution in the use of AI for early breast cancer (BC) diagnosis, dividing the research timeline into two periods: 2012–2022 and 2023–Q1 2025. In both periods, “breast cancer” remains the dominant theme, highlighting its sustained central role in the field.

During the earlier period, the literature focused on core AI techniques such as traditional machine learning models, deep learning, segmentation, and feature extraction. This phase marks the initial adoption of AI in the field and laid the foundation for subsequent advancements.

In the more recent period (2023–2025), the focus has shifted toward specialized and clinically oriented applications. Research now emphasizes advanced segmentation techniques like U-Net, alongside technologies including computer-aided diagnosis (CAD), convolutional neural networks (CNN), digital mammography, and tomosynthesis. Additionally, the appearance of terms such as “risk factors” and performance metrics signals a growing emphasis on clinical relevance and diagnostic accuracy.

Overall, Fig. 10 demonstrates how keyword prominence has evolved over time. Terms gaining influence have moved closer to the center of the thematic map, while others have shifted toward the periphery, indicating either declining focus or more specialized, narrower research areas.

## Discussion

The conceptual foundations of artificial intelligence (AI) emerged in the mid-20th century with pivotal contributions such as McCulloch and Pitts’ binary neuron model, Rosenblatt’s perceptron architecture, Shannon’s information theory, and Turing’s formulation of the Turing Test [17, 18, 19, 20]. These theoretical breakthroughs laid the groundwork for early rule-based systems, culminating in medical tools like MYCIN in the 1970s—among the first AI systems used in clinical decision-making [6].

The application of AI to early breast cancer (BC) diagnosis first appeared in academic literature in 1994, in a study by Susannah K. Rogers from the University of Texas at Austin. This article introduced artificial neural networks as tools capable of learning and generalizing, with the potential to reduce false positives in diagnostic imaging [21]. Wu’s 1995 work further advanced this direction by applying neural networks to the classification of microcalcifications [22]. From 1994 to 2012, only 45 relevant articles were published, nearly half originating from the United States, indicating a nascent research phase.

A clear turning point occurred in 2012 with the increasing application of convolutional neural networks (CNNs) and deep learning techniques to diagnostic imaging. This period coincided with technological advances such as GPU acceleration and cloud computing, which facilitated broader experimentation and clinical integration. Our bibliometric analysis highlights this pivotal transformation, mapping not only growth patterns and institutional engagement but also the conceptual shift toward personalized, algorithm-driven diagnostics.

Recent advances show that AI models are increasingly applied in clinical practice. For example, AI systems in mammography have improved cancer detection and reduced false positives in large trials [23]. In ultrasound, AI aids breast nodule classification, enhancing diagnosis and reducing unnecessary biopsies [24]. Beyond imaging, AI models integrating clinical and genomic data help predict treatment benefits and recurrence risk in breast cancer patients [25]. These examples highlight AI’s role in improving diagnosis and supporting personalized treatment.

Looking ahead, the integration of AI into standard clinical workflows will likely intensify. While further validation is essential, human-dependent imaging techniques—such as ultrasound—may increasingly be enhanced by AI-guided robotic platforms, potentially offering greater consistency and efficiency in image acquisition [26]. Additionally, automated reporting systems are expected to become standardized, streamlining radiological assessments. These developments point to an evolving role for AI—not only as a diagnostic aid but also as a potential lead actor in clinical decision-making.

Beyond imaging, AI is anticipated to improve histopathological interpretation, enable more accurate risk stratification, and support personalized treatment planning. Progress in genomic sequencing, gene expression profiling, and mutation analysis will allow for real-time decision support based on genetic risk scores [27]. As these technologies expand, ethical considerations—such as algorithmic transparency, data privacy, and equitable access—must be addressed to ensure responsible clinical adoption.

While several bibliometric studies have investigated AI’s role in breast cancer, none has focused as comprehensively on early diagnosis or covered the field with the methodological breadth and temporal scope presented here. For example, Zhang et al. [28] employed topic modeling on 511 Web of Science-indexed articles from 2000 to 2021 but did not capture the sharp increase in publications after 2021, nor did they explore collaboration networks. Salod and Singh [29] analyzed PubMed articles between 2015 and 2019, focusing narrowly on machine learning without cross-database synthesis. Hou et al. [30] emphasized multimodal imaging yet overlooked emergent areas such as AI-driven biomarker detection and genetic profiling. Likewise, Syed and Khan [31] offered a broad overview of AI in oncology but did not explore thematic evolution or co-word networks in depth.

In contrast, our study integrates data from both Scopus and Web of Science, covers publications through the first quarter of 2025, and focuses specifically on AI applications in early BC detection—a clinically critical but underexplored area. In addition to traditional metrics such as citation counts and author productivity, we employed co-authorship, co-citation, and co-word analyses to identify emerging themes and collaborative patterns, thereby capturing the field’s intellectual structure. These elements not only extend prior literature but also provide a forward-looking roadmap for future research and clinical innovation.

Importantly, our findings also reveal distinct national research dynamics. China and India lead in publication volume, while the United States and Greece stand out in citation impact. These discrepancies may reflect differences in research funding, focus, and collaboration models. For example, China’s rapid growth has been enabled by government-supported AI initiatives and an emphasis on data infrastructure within healthcare strategies [32], while India’s expanding publication base reflects rising interest in digital health despite persistent challenges in infrastructure, governance, and equity of access [33]. In contrast, higher citation impact in countries like the United States and Greece is broadly consistent with evidence that international collaboration correlates with greater citation impact and global visibility [34]. These patterns suggest that while publication volume signals investment and workforce capacity, citation impact reflects scientific influence and translational relevance. Encouraging balanced international collaborations may help high-output countries convert productivity into broader clinical and scientific value.

In summary, this study provides more than a descriptive mapping of existing literature; it offers a strategic lens through which to interpret the developmental trajectory of AI in early breast cancer diagnosis. By uncovering conceptual shifts, collaborative patterns, and underexplored subtopics, the analysis supports not only retrospective understanding but also future-oriented thinking. The findings may guide researchers in identifying research gaps, assist policymakers in recognizing innovation trends, and help clinicians understand how bibliometric signals align with technological adoption. In this way, the study bridges the divide between bibliometric knowledge and real-world clinical potential—positioning early detection as a dynamic, data-driven frontier in cancer care.

## Limitations

Like many bibliometric studies, this analysis has certain limitations. Bibliometric methods effectively identify publication trends, citation dynamics, and collaboration patterns, but they do not evaluate the methodological quality or clinical impact of individual studies.

Relying solely on the Web of Science and Scopus databases may have excluded potentially relevant records from other sources such as PubMed or IEEE Xplore. However, these two databases were selected for their comprehensive interdisciplinary coverage, structured metadata, and compatibility with bibliometric analysis tools, which ensures data consistency and comparability.

Additionally, although the dataset includes publications up to the first quarter of 2025, the bibliometric landscape is continuously evolving. Future studies could benefit from periodically updating the data to reflect emerging contributions and shifting trends beyond this timeframe.

Finally, while the search strategy was transparently constructed and the Boolean query explicitly shared in the Methods section, the reproducibility of bibliometric workflows may still be affected by database indexing variations and software-specific limitations during co-authorship, co-citation, and thematic mapping analyses. These technical nuances can influence the clustering outputs and should therefore be interpreted within the constraints of these technical limitations.

## Data Availability

The data analysed in this study were obtained from Web of Science and Scopus databases. These databases are subscription-based and accessible via institutional access. Data can be shared upon reasonable request to the corresponding author.
